# Unusual insidious spinal accessory nerve palsy: a case report

**DOI:** 10.1186/1752-1947-4-158

**Published:** 2010-05-27

**Authors:** Ioannis N Charopoulos, Nikolas Hadjinicolaou, Ioannis Aktselis, George P Lyritis, Nikolaos Papaioannou, Constantinos Kokoroghiannis

**Affiliations:** 1 Fifth Orthopedic Department, KAT Hospital, 14561, Greece; 2 Laboratory for Musculoskeletal System Research, Medical School, University of Athens, Greece

## Abstract

**Introduction:**

Isolated spinal accessory nerve dysfunction has a major detrimental impact on the functional performance of the shoulder girdle, and is a well-documented complication of surgical procedures in the posterior triangle of the neck. To the best of our knowledge, the natural course and the most effective way of handling spontaneous spinal accessory nerve palsy has been described in only a few instances in the literature.

**Case presentation:**

We report the case of a 36-year-old Caucasian, Greek man with spontaneous unilateral trapezius palsy with an insidious course. To the best of our knowledge, few such cases have been documented in the literature. The unusual clinical presentation and functional performance mismatch with the imaging findings were also observed. Our patient showed a deterioration that was different from the usual course of this pathology, with an early onset of irreversible trapezius muscle dysfunction two months after the first clinical signs started to manifest. A surgical reconstruction was proposed as the most efficient treatment, but our patient declined this. Although he failed to recover fully after conservative treatment for eight months, he regained moderate function and is currently virtually pain-free.

**Conclusion:**

Clinicians have to be aware that due to anatomical variation and the potential for compensation by the levator scapulae, the clinical consequences of any injury to the spinal accessory nerve may vary.

## Introduction

Isolated spinal accessory nerve dysfunction has a serious impact on the functional performance of the shoulder girdle. The role of the trapezius muscle in shoulder girdle kinesiology is fundamental, since it contributes to the scapulothoracic rhythm by elevating, rotating and retracting the scapula. Although spinal accessory nerve palsy is a well-documented complication of surgical procedures in the posterior triangle of the neck [1,2], several other possible causes have been proposed [3-6].

The usual initial presentation is that of severe neck and shoulder pain, sometimes radiating to the arm but without the initial palsy [7-9]. The isolated spinal accessory neuropathy usually becomes evident after a few days, with weakness in the abduction and anterior elevation of the arm, and with atrophy of the trapezius muscle and winging of the scapula after a few weeks. Occasional variations in the clinical findings of patients with identical lesions of the spinal accessory nerve may be partially explained by variations in the innervations of the trapezius muscle [7].

It is essential to recognize the condition and its variants promptly and in the early stage so as to avoid the reduction of scapulothoracic motion which occurred with our patient. We report a case of spontaneous unilateral trapezius palsy with an insidious course, which has been documented in only a few instances in the literature [3,8,9]. Moreover, parameters such as our patient's unusual initial clinical presentation, the magnitude of the functional deficit and its mismatch with the imaging and electrophysiological findings, as well as a possible pathomechanism of the present injury, are discussed in this case report.

## Case presentation

A 36-year-old Caucasian, Greek man presented to the out-patient clinic of KAT hospital complaining of weakness and limited range of motion of his right shoulder. He had noticed, over the past two months, that abduction and elevation of the joint had gradually become limited while he was carrying his newborn child in a baby basket. He denied any neck or shoulder pain and could not recall a specific precipitating traumatic event or any recent episode of respiratory infection. However, he reported that his job was a heavy manual one, requiring lifting and carrying heavy objects on his shoulders. Our patient had no significant medical history.

**Figure 1:**
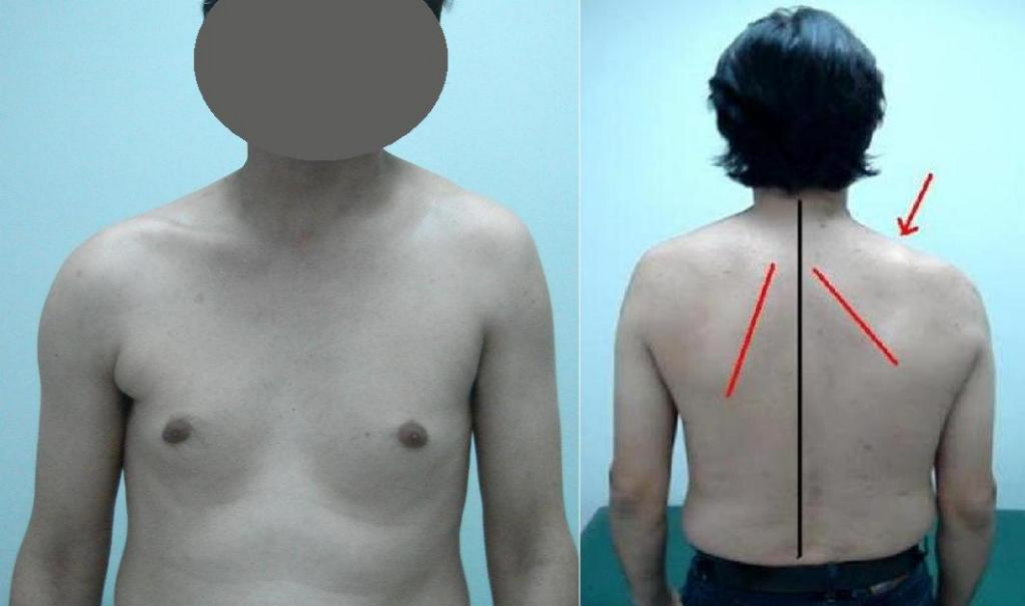
**Frontal and dorsal views demonstrating the right neck asymmetry and ipsilateral shoulder depression (red arrow)**. The right scapula's medial wall (red line) is translated laterally, as it is evident with its increased distance from the body's midline (black line).

**Figure 2:**
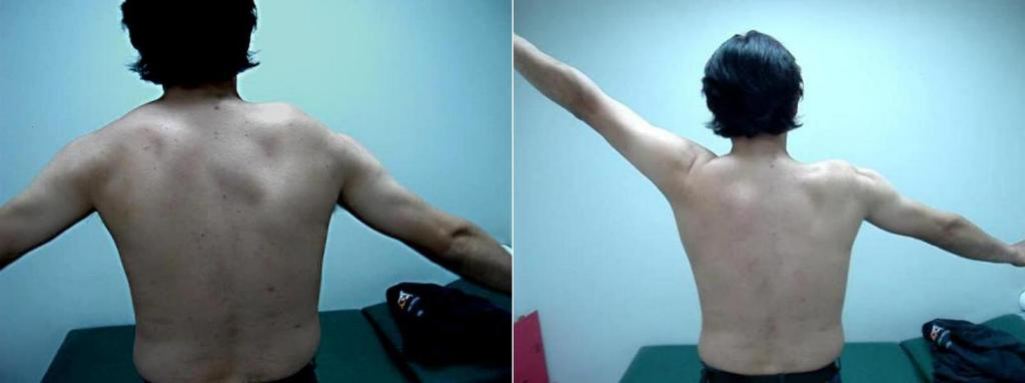
**Scapular winging at different angles of arm abduction**.

Physical examination revealed a winged scapula and asymmetry of his shoulders, with right shoulder depression (Figure 1). He was unable to abduct his right arm above 80° in the frontal or scapular plane while his forward elevation was slightly reduced. His passive range of motion was comparable to the normal left side. Scapular winging was marked during abduction and disappeared in forward elevation, while it was only slightly evident at rest (Figure 2). Furthermore, there was a marked wasting of his right trapezius muscle with decreased shrugging of the affected shoulder. Both wasting and weakness were not observed in the ipsilateral sternocleidomastoid muscle, and a neurological examination did not reveal other cranial nerve deficits. No brachial plexus neurological signs were detected. Our patient's rotator cuff was judged to be intact.

**Figure 3:**
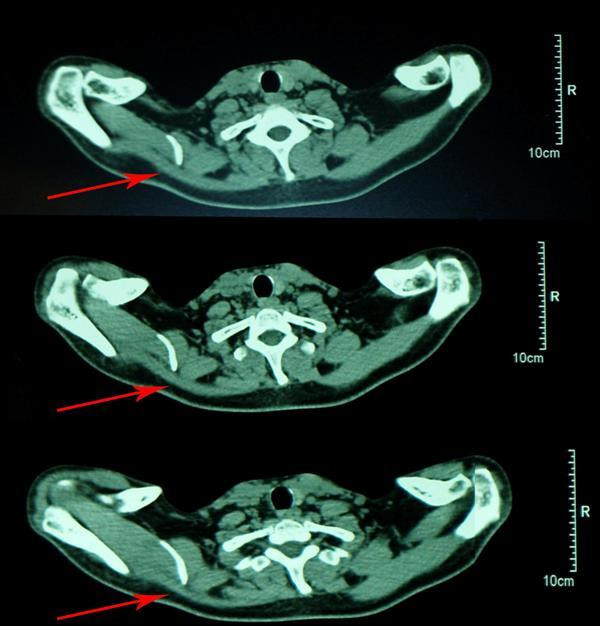
**Transverse views of computed tomography scans reveal marked wasting in the muscle bulk of almost all parts of the right trapezius (arrows)**.

**Figure 4:**
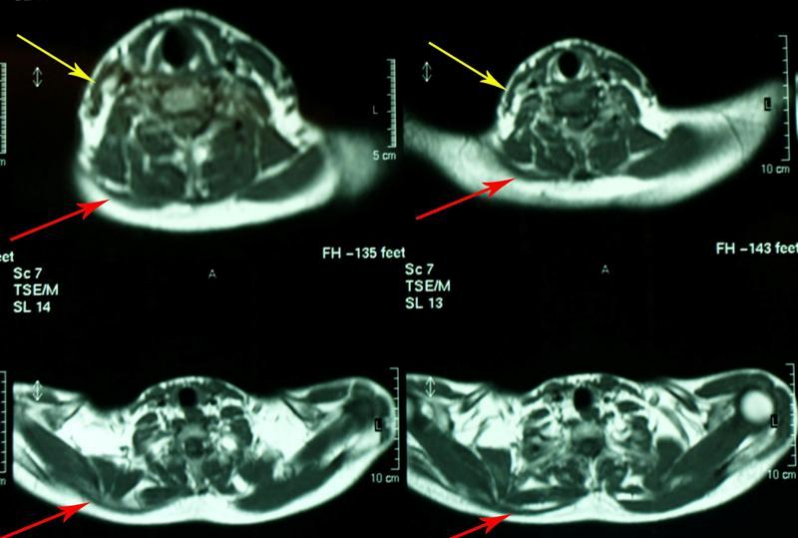
**Magnetic resonance imaging of the neck showing muscle wasting of the right trapezius (red arrows) and sternocleidomastoid (yellow arrows) muscles**.

Results of his complete blood count, erythrocyte sedimentation rate (ESR), C-reactive protein (CRP), and serum biochemistry were all normal. The initial X-rays of his right shoulder region were unremarkable. Computed tomography (CT) of the shoulder girdle of our patient revealed significant diffuse trapezius muscle wasting (Figure 3). Magnetic resonance imaging (MRI) of his cervical spine, right shoulder joint and skull base identified no pathology except mild atrophy of his right sternocleidomastoid and trapezius muscles (Figure 4).

Nerve conduction study of our patient's right spinal accessory nerve, with surface stimulation along the posterior border of the sternocleidomastoid muscle and recording from the trapezius, produced no compound muscle action potential. A needle electromyography (EMG) of the right trapezius muscle revealed signs of active denervation (fibrillation potentials and positive sharp waves), atrophy (marked diminution of insertional activity), and severe axonal damage (no recruitment of motor units). Meanwhile, EMG of his right sternocleidomastoid muscle showed findings suggestive of mild axonal injury (decreased recruitment, polyphasicity and prolonged duration with increased amplitude of motor unit potentials). His levator scapulae, serratus anterior and rhomboid muscles were normal. Electrophysiological findings suggested an axonal degeneration of his right spinal accessory nerve that was mainly distal to the innervation of the sternocleidomastoid muscle, and an irreversible denervation of his right trapezius muscle. Consequently, a diagnosis of spontaneous chronic right spinal accessory nerve palsy was established, although not affecting some fibers to the sternocleidomastoid muscle.

The chronic nature of our patient's lesion and the denervation of his trapezius muscle with severe loss of most of its motor units suggested the appropriate treatment procedure was dynamic muscle transfer using the levator scapulae and the rhomboid muscles. Our patient refused the recommended treatment, since he felt that his painless disability did not justify this highly demanding procedure. Instead he followed a specific program of physiotherapy, focusing on resistance exercises to progressively strengthen the adjacent scapular muscles and on exercises to preserve the maximum range of motion of his shoulder joint.

Thereafter, he was followed up every month for assessing and modulating the progress of his rehabilitation program and for subsequent EMG investigations. At the last follow-up, eight months after the onset, a slight improvement in the active abduction of his arm and in his neck asymmetry was observed. Significantly, his scapular winging remained painless, with no associated neurological deficits. Our patient was also able to perform his manual work, at approximately the same level as before. A repeat EMG did not show any alterations from the initial electrophysiological findings.

## Discussion

The role of the trapezius muscle in shoulder girdle kinesiology is fundamental, since it supports the entire weight of the upper extremity in the erect position, along with the levator scapulae muscle. Moreover, its middle portion is the initiator of upward rotation of the scapula, while its upper and lower portions elevate the lateral angle of the scapula and pull down the medial edge of the scapular spine [10]. Also, shrugging of the shoulder and retraction of the scapula rely mainly on this muscle. In summary, it contributes to the scapulothoracic rhythm by elevating, rotating and retracting the scapula.

Trapezius muscle dysfunction causes drooping of the shoulder, asymmetry of the neckline, winging of the scapula, and weakness of forward elevation and abduction movements. Furthermore, the intricate balance of muscle forces about the scapula is disrupted and the smoothness of the scapulohumeral rhythm is lost. Concerning scapular winging, the scapula assumes a depressed and lateral translated position, while the inferior scapular angle rotates laterally [4] (Figure 1). Therefore, this lesion can be not only painful but also deforming and disabling [2,3]. The pain that develops can be quite severe because of muscle spasm, radiculitis from traction on the brachial plexus, frozen shoulder, or subacromial impingement. Aching may radiate to the medial margin of the scapula and down the arm to the fingers, and is also sometimes incapacitating [7].

In our case report, we illustrate spinal accessory nerve palsy of spontaneous insidious onset, which has been described in only a few instances in the literature [1,7-9,11,12]. Although our case demonstrated common clinical signs of this pathology, we have observed certain unique characteristics of patient. First, his main concerns and complaints were right shoulder weakness and decreased active range of motion, and not pain or neurological symptoms which are mostly reported in the literature. Furthermore, severe trapezius muscle dysfunction, as assessed by EMG, revealed that the spinal accessory nerve dysfunction of our patient must have pre-existed before becoming clinically apparent. In other words, this latency period supports our hypothesis of the insidious nature of the lesion and our view that it eventually emerged when the compensatory mechanisms were exhausted.

The initial painless clinical presentation, along with the limited functional deficit even after eight months, were not consistent with our imaging and electrophysiological findings. The extent of the trapezius muscle atrophy could have produced a gamut of symptoms including radiculitis, restriction of passive shoulder motion, and impingement. By contrast, this case shows unusual features with constant discomfort due to neckline asymmetry and restricted arm abduction. The compensatory action of the other scapular stabilizers seems to explain this inconsistency.

The mild handicaps and good results after conservative treatment reported in other cases of spontaneous onset [7,8,11,12] did not apply in our case. Our patient showed irreversible deterioration of his trapezius muscle function quite early, two months after the appearance of his first clinical signs, which was different from the usual outcome of such a lesion. The massive trapezius muscle atrophy with the severe loss of most of its motor units seemed to exclude the usually proposed surgical procedures of neurolysis and nerve grafting [13,14]. However, there are reports of poor results after microsurgical repair of nerves in cases of spontaneous trapezius palsy [1]. Although transfer of the levator scapulae and the rhomboids to substitute for the three components of the trapezius muscle appeared to be the most appropriate treatment, our patient declined it because he was pain-free and willing to accept his functional disability. Our patient failed to make a full recovery after conservative treatment for eight months. He regained moderate function and was virtually pain-free.

The main cause of trapezius palsy is injury to its major nerve supply, the spinal accessory nerve. The superficial location of the spinal accessory nerve, in the subcutaneous tissue on the floor of the posterior cervical triangle makes it vulnerable to injury [2]. This palsy is commonly seen after surgical procedures in the posterior cervical triangle for malignant diseases and after penetrating injuries [1,2]. Other reported mechanisms of injury include blunt trauma or a direct blow in the neck region [2,5], compression by tumors at the base of the skull [6], fractures involving the jugular foramen [4], and stretching of the nerve after depression of the shoulder with the head being forced in the opposite direction [12]. Isolated rare causes that have been reported are aneurysm formation, whiplash injury, acromioclavicular or sternoclavicular dislocation and catheterization of the internal jugular vein [1].

Long-standing heavy manual work, including carrying heavy objects on the shoulder, seem to have been be the precipitating factor for the spontaneous insidious onset of trapezius palsy in our patient. The ensuing repetitive microtrauma caused localized spinal accessory nerve compression and subsequent aseptic inflammation, which caused deterioration in the trapezius muscle. This hypothesis justifies the insidious and chronic nature of the observed functional deficit.

Electrophysiological findings revealed greater dysfunction of the trapezius muscle relative to the ipsilateral sternocleidomastoid muscle. The vulnerability of the spinal accessory nerve fibers supplying the trapezius muscle selectively could be explained by their superficial anatomic location in the posterior cervical triangle, just caudal to the branch for the sternocleidomastoid muscle. The lesser severity of the electrophysiological changes obtained for the nerve fibers innervating the sternocleidomastoid muscle could be explained by the deeper anatomic location of the specific nerve branch and possibly by the spatial topographic distribution of the fibers in the spinal accessory nerve. This is quite ambiguous since it is not well discussed in the relevant literature.

Idiopathic isolated spinal accessory palsy should have been considered in the differential diagnosis of our patient, since similar cases have been reported [8,15]. Distinguishing neuralgic amyotrophy from gradual compression palsy, based solely on presenting symptoms and clinical and electrophysiological examinations, is quite challenging. Furthermore, neither pain characteristics nor the resultant weaknesses can distinguish these two causes [15]. In the current report, the insidious course of the deficit, the lack of pain at the initial presentation and the relatively sparing of sternocleidomastoid nerve fibers favour localized nerve compression over neuralgic amyotrophy as the most probable cause.

## Conclusions

The clinical relevance of our case report focuses on the significance of the hierarchical clinical evaluation of the shoulder girdle kinesiology. In particular, we highlight the significance of clinical examination assisted by the findings of different imaging modalities and the use of EMG in assessing the broad spectrum of causes of scapulothoracic dyskinesia. It is stressed that the dynamic motion of the scapulothoracic articulation should be evaluated as a co-ordinated movement of the muscle units of the shoulder. Therefore, in order to plan treatment, clinicians should evaluate each unit of scapulothoracic motion separately and should be aware that anatomical variations and the potential for compensation by the levator scapulae may cause the clinical consequences from any injury to the spinal accessory nerve to differ.

## Competing interests

The authors declare that they have no competing interests.

## Authors' contributions

INC co-ordinated the diagnostic and therapeutic approach and conceived the idea of presenting the case report. NC and IA assisted in the sequential imaging control and in the preparation and drafting of the manuscript. CK assisted in the drafting of the manuscript. NP and CK made the final check and approval of the submitted manuscript. All the authors read and approved the final manuscript.

## Consent

Written informed consent was obtained from the patient for publication of this case report and any accompanying images. A copy of the written consent is available for review by the Editor-in-Chief of this journal.
